# Value of Early Kinetics of Procalcitonin with Point-of-Care Test to Predict Postoperative Abscess Following Non-Complicated Acute Appendicitis: A Pilot Study

**DOI:** 10.3390/medicina61081374

**Published:** 2025-07-29

**Authors:** Pietro Fransvea, Valeria Fico, Claudia Arcangeli, Gaia Altieri, Giuseppe Tropeano, Marta Di Grezia, Gilda Pepe, Filomena Misuriello, Giuseppe Brisinda, Gabriele Sganga, Sergio Alfieri

**Affiliations:** 1 Emergency Surgery and Trauma Unit, Department of Medical and Surgical Sciences, Fondazione Policlinico Universitario A Gemelli, IRCCS, 00168 Rome, Italy; pietro.fransvea@policlinicogemelli.it (P.F.); valeria.fico@policlinicogemelli.it (V.F.); gaia.altieri@guest.policlinicogemelli.it (G.A.); giuseppe.tropeano@guest.policlinicogemelli.it (G.T.); marta.digrezia@policlinicogemelli.it (M.D.G.); gilda.pepe@policlinicogemelli.it (G.P.); sergio.alfieri@policlinicogemelli.it (S.A.); 2 Department of Translational Medicine and Surgery, Catholic School of Medicine “Agostino Gemelli”, 00168 Rome, Italy; arcangeli.claudia@hsr.it (C.A.); filomena.misuriello01@icatt.it (F.M.); gabriele.sganga@policlinicogemelli.it (G.S.); 3 Digestive Surgery Unit, Department of Medical and Surgical Sciences, Fondazione Policlinico Universitario A Gemelli, IRCCS, 00168 Rome, Italy

**Keywords:** artificial intelligence, acute care surgery, acute appendicitis, procalcitonin

## Abstract

*Background and Objectives:* Acute appendicitis is a common surgical emergency, and while appendectomy typically results in good outcomes, post-operative complications, like intra-abdominal abscesses, can occur. Traditional biomarkers, such as white blood cells count and C-reactive protein, often lack the accuracy needed for early detection. Procalcitonin is emerging as a potential marker for predicting post-operative infections. This pilot study evaluates the role of kinetics of procalcitonin, measured via point-of-care testing, in predicting abscess formation in patients with non-complicated appendicitis. *Materials and Methods:* The study involved 33 patients undergoing appendectomy for non-complicated acute appendicitis. The levels of procalcitonin were measured at four time points: pre-operatively (T0), post-operatively (T1), on the first post-operative day (T2), and at discharge (T3). The primary outcome was the development of post-operative abscesses, confirmed by imaging or intervention. *Results:* Four patients (12%) developed abscesses. The levels of procalcitonin were significantly higher in the abscess group at all time points compared to the non-abscess group (*p* < 0.05). The levels of procalcitonin in the abscess group plateaued after an initial post-operative decline, while levels steadily decreased in the non-abscess group. *Conclusions:* Procalcitonin, particularly its kinetic profile, may serve as a valuable early marker for predicting post-operative abscess formation. Point-of-care testing for procalcitonin can enable timely intervention, improving outcomes. Kinetics of procalcitonin show promise as a predictor for post-operative abscesses after appendectomy, though larger studies are needed to confirm these findings.

## 1. Introduction

Acute appendicitis is one of the most frequent causes of emergency abdominal surgery worldwide, with an incidence of approximately 100 cases per 100,000 individuals annually, and it often necessitates prompt surgical intervention to prevent complications such as perforation or peritonitis [1,2,3]. Despite being considered a standard and relatively low-risk procedure, appendectomy is not devoid of morbidity. In particular, post-operative complications such as intra-abdominal abscesses remain a clinically relevant issue, even in patients with apparently non-complicated forms of the disease [4,5]. These abscesses can significantly prolong hospital stay, increase healthcare costs, and lead to further interventions such as reoperation or radiologically guided drainage, in addition to prolonged antibiotic use [6,7,8].

Recent improvements in diagnostic accuracy, including advanced imaging modalities (e.g., high-resolution ultrasound, contrast-enhanced CT), have enhanced the clinician’s ability to diagnose appendicitis early and to stratify patients based on severity. Alongside imaging, laboratory parameters such as white blood cell (WBC) count, C-reactive protein (CRP), and bilirubin are widely used to support diagnostic reasoning and to monitor clinical progression [9]. Nevertheless, their utility in predicting post-operative infectious complications remains limited due to insufficient specificity and delayed response kinetics.

Procalcitonin (PCT), a 116-amino acid prohormone of calcitonin, has gained increasing attention in recent years as a promising biomarker for bacterial infections, given its rapid response to endotoxins and pro-inflammatory cytokines such as IL-6 and TNF-alpha [10,11,12,13,14]. Unlike CRP, which rises in various inflammatory states, PCT exhibits a higher specificity for bacterial infections and sepsis, making it particularly valuable in the early detection of clinically significant infectious complications.

The potential of PCT as a predictive marker extends beyond diagnosis: several studies have demonstrated its prognostic value in a wide range of surgical settings, including colorectal surgery, pancreatic resections, and treatment of intra-abdominal sepsis. Despite these promising findings, there is still a paucity of data specifically evaluating the role of PCT kinetics in the post-operative course of patients with non-complicated appendicitis—a subgroup traditionally considered at low risk for infectious sequelae but in which abscess formation can still occur.

In this regard, the use of point-of-care testing (POCT) for PCT offers a novel advantage. This approach allows for rapid, bedside measurement of serum PCT concentrations, enabling clinicians to make time-sensitive decisions based on dynamic changes in biomarker levels rather than relying solely on static thresholds. Moreover, POCT is especially advantageous in emergency settings, where early identification of patients at risk for complications can significantly impact management strategies and resource allocation. The incorporation of PCT kinetics into standardized post-operative protocols could, thus, serve not only as a tool for early diagnosis of infection but also as a guide for therapeutic decision-making, including the initiation or escalation of antibiotic therapy, radiologic imaging, or closer clinical observation.

This pilot study was, therefore, designed to investigate whether dynamic changes in PCT, measured at multiple time points through both traditional laboratory techniques and POCT, can serve as early predictors of intra-abdominal abscess formation following appendectomy for non-complicated appendicitis. To ensure methodological consistency, all surgical procedures were performed by either attending surgeons or senior residents under direct supervision, following a standardized operative protocol. Laparoscopic procedures were carried out using a three-trocar technique with end-loop or stapler closure of the appendiceal stump, while open appendectomies were reserved for specific indications and performed through a McBurney or Rocky–Davis incision. Post-operative monitoring included serial measurements of serum PCT at pre-defined intervals, comprehensive clinical assessment, and abdominal imaging in cases of suspected complications. Abscesses were radiologically confirmed via CT or ultrasound as collections of fluid with or without gas, and those requiring intervention were managed with CT-guided percutaneous drainage performed by experienced interventional radiologists using pigtail catheters under local anaesthesia. Antibiotic therapy was initiated empirically and adjusted based on microbiological cultures when available. Finally, statistical analysis, including ROC curve assessment, was performed to evaluate the diagnostic accuracy and discriminative capacity of PCT values and their kinetics in identifying patients at risk for abscess formation.

## 2. Materials and Methods

This single-centre, prospective observational cohort study was conducted at the Fondazione Policlinico Universitario A. Gemelli IRCCS in Rome, Italy, over a two-year period, from 1 January 2022 to 31 December 2023. The study aimed to investigate the prognostic value of procalcitonin (PCT) kinetics in predicting post-operative intra-abdominal abscesses following appendectomy for non-complicated acute appendicitis.

All adult patients (aged > 18 years) who presented to the emergency department with a diagnosis of acute appendicitis and subsequently underwent emergency appendectomy—whether performed laparoscopically or via open surgery—were screened for potential inclusion. Only patients diagnosed with uncomplicated acute appendicitis and able to provide written informed consent were considered eligible.

Patients were excluded in cases of pre-existing intra-abdominal abscess, complicated appendicitis at the time of diagnosis (e.g., perforation, peritonitis, phlegmon), pregnancy, chronic pulmonary diseases, or if they had received antibiotic therapy prior to hospital admission. For each enrolled patient, a standardized data collection protocol was implemented.

Demographic variables (age, sex), clinical features (pain characteristics, duration of symptoms), and relevant comorbidities (including arterial hypertension, diabetes, and other chronic conditions) were recorded. Baseline and post-operative laboratory parameters were obtained, including white blood cell count (WBC), C-reactive protein (CRP), bilirubin, creatinine, and glycemia.

Particular attention was given to PCT, which was measured at four defined time points to assess its dynamic profile: upon arrival at the emergency department before surgery (T0), immediately after the surgical procedure (T1), on the first post-operative day (T2), and at the time of discharge (T3). PCT levels were determined via venous blood sampling and quantified using an electrochemiluminescence immunoassay, with values below 0.05 ng/mL considered negative. In addition, point-of-care testing (POCT) was conducted using the BRAHMS PCT-Q system (Thermo-Fisher Scientific), a semi-quantitative lateral flow immunoassay capable of detecting serum PCT concentrations ranging from 0.05 to 200 ng/mL. POCT allowed for rapid results, available within approximately 30 min, thereby facilitating timely clinical decision-making.

The surgical procedures were performed according to a standardized technique. Laparoscopic appendectomies were carried out using a three-trocar approach with either end-loop or stapler stump closure, while open surgeries were performed through a right lower quadrant incision.

Post-operative clinical monitoring included routine laboratory evaluation and physical examination. Radiological investigations (ultrasound or contrast-enhanced CT scan) were employed in patients exhibiting clinical signs suggestive of infection. Intra-abdominal abscesses were defined as fluid collections identified on imaging, with or without gas formation, requiring therapeutic intervention such as percutaneous drainage or extended antibiotic therapy. Drainage procedures were executed under CT guidance by interventional radiologists using pigtail catheters (10–12 Fr) placed under local anaesthesia and aseptic technique.

Patients were stratified into two groups for analysis: those who developed post-operative intra-abdominal abscesses (Group 1) and those who did not (Group 2). All patients completed the full sequence of PCT measurements and were included in the final analysis.

Data were expressed as mean ± standard deviation (SD) or as percentages where appropriate. Comparisons between groups were conducted using Student’s t-test for continuous variables and Pearson’s chi-square test for categorical data. Where necessary, analysis of variance (ANOVA) was employed to assess differences across time points. Multivariate logistic regression was used to identify independent predictors of abscess development, adjusting for potential confounders. The predictive performance of PCT was further assessed using receiver operating characteristic (ROC) curve analysis, with particular attention to values at T2 (first post-operative day), where the area under the curve (AUC) reached 0.88, indicating high diagnostic accuracy. Statistical significance was defined as a *p*-value less than 0.05. All analyses were conducted using SPSS 29 software (IBM Corp., Armonk, NY, USA).

## 3. Results

A total of 33 patients were enrolled in this pilot study. The baseline data were reported in Table 1.

Most patients (97.0%, 33 patients) underwent laparoscopic appendectomy, with an average hospital stay of 3.0 ± 1.0 days.

There were no recorded deaths, and the overall morbidity rate was 27.3%, primarily categorized as Clavien–Dindo grade I complications [15].

PCT values in all patients at different dosing times are reported in Table 2.

Four patients (12.1%) developed an intra-abdominal abscess. The mean time to abscess formation was 6.0 ± 1.8 days. The mean hospital stay for abscess-related re-admissions was 9.0 ± 2.1 days. Three patients required CT-guided percutaneous drainage, and one patient was managed with broad-spectrum antibiotics alone.

The comparison of clinical and laboratory data in the two groups of patients (Group 1—Abscess and Group 2—No abscess) is reported in Table 3.

The PCT levels in the abscess group (Group 1) were significantly higher compared to the control group (Group 2) at all time points. At T0, Group 1 had a mean PCT of 2.92 ± 4.5 ng/mL, compared to 0.56 ± 0.80 ng/mL in Group 2 (*p* = 0.01). Similarly, at T1, Group 1 showed a mean PCT of 1.49 ± 1.5 ng/mL, significantly higher than the control group’s 0.26 ± 0.51 ng/mL (*p* = 0.003). PCT levels remained elevated in Group 1 at T2 (1.40 ± 0.9 ng/mL vs. 0.26 ± 0.25 ng/mL, *p* = 0.001) and T3 (0.64 ± 0.74 ng/mL vs. 0.15 ± 0.25 ng/mL, *p* = 0.006).

**Figure 1:**
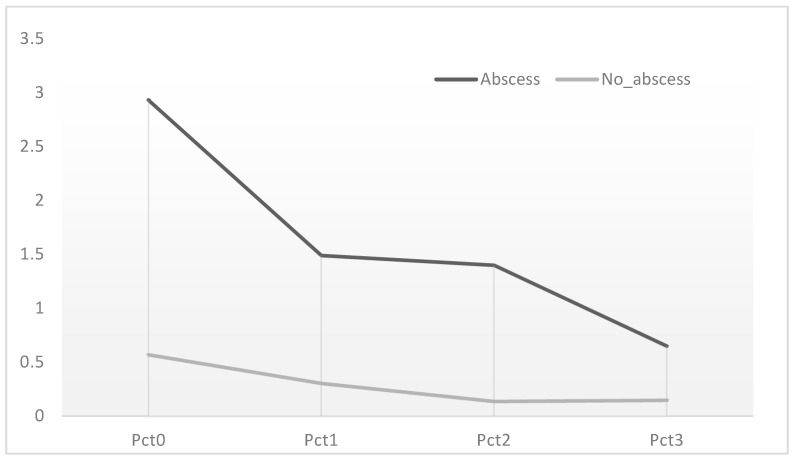
PCT kinetics between the groups. Graphic of PCT values at mean PCT values at time 0, 1, 2, 3.

In both groups, POCT measurements closely paralleled those obtained from traditional blood draws, showing strong correlation. In the abscess group, PCT levels initially declined but plateaued after T1, while in the non-abscess group, levels consistently decreased over time (Figure 1; Table 4).

Furthermore, it is worth noting that the observed plateauing of PCT levels in patients who developed abscesses may reflect an ongoing localized infectious process insufficiently resolved by initial surgical intervention. This physiological response supports the concept that PCT not only indicates infection presence but also correlates with the persistence or resolution of the underlying inflammatory condition. In surgical decision-making, such data could be pivotal in stratifying patients postoperatively: those requiring only observation versus those warranting early imaging and possibly preemptive treatment.

From a healthcare management perspective, early identification of patients at risk of complications could reduce re-hospitalization rates, shorten lengths of stay, and optimize resource allocation, particularly in high-volume emergency surgical settings.

Given that three out of four patients in our study required interventional radiology procedures, earlier recognition through PCT trends could potentially alter their clinical course. Thus, the integration of PCT kinetics into routine postoperative monitoring protocols deserves strong consideration, especially when validated by future large-scale studies.

## 4. Discussion

Our pilot study investigated the role of serum procalcitonin (PCT) and its dynamic changes as a potential early biomarker for predicting post-operative intra-abdominal abscesses following appendectomy for non-complicated acute appendicitis.

Acute appendicitis remains the most frequent cause of emergency abdominal surgery worldwide, with surgery still considered the standard of care despite growing interest in non-operative management for selected patients [16,17,18,19,20,21]. Although non-operative strategies have demonstrated safety and feasibility, especially in uncomplicated cases, recurrence rates of up to 39% within five years support the continued preference for appendectomy as definitive treatment [21]. Post-operative intra-abdominal abscesses, although not common, are the most frequent surgical complication, with an incidence ranging between 1% and 10% in all patients, and around 1.9% specifically in uncomplicated cases [22,23]. These complications significantly prolong hospital stay, increase healthcare costs, and may require additional interventions such as CT-guided drainage or prolonged antibiotic therapy.

Several intraoperative factors have been evaluated in the literature as potential contributors to abscess formation, including methods of stump closure [24,25,26], the routine use of intra-abdominal drains [27,28,29], and peritoneal irrigation [30,31,32]. However, to date, only complicated appendicitis has been consistently identified as an independent risk factor for post-operative abscess development [1,23]. Nevertheless, as confirmed in our cohort, abscesses can still occur following procedures for uncomplicated appendicitis, albeit at lower rates.

The ability to identify patients at risk early in the post-operative course could, therefore, allow timely interventions, minimizing the clinical and economic burden of these complications. In our study, patients who developed abscesses required an average of 9.0 ± 2.1 additional hospital days and three out of four underwent CT-guided percutaneous drainage, highlighting the tangible impact of such complications. In recent years, there has been increasing interest in identifying serum biomarkers capable of predicting post-operative infectious complications. Traditional inflammatory markers such as WBC and CRP have limited predictive accuracy in this context [33,34]. Conversely, PCT has been shown to be a sensitive and specific marker for bacterial infections and sepsis [35]. Synthesized by C-cells of the thyroid and various other tissues in response to bacterial toxins, PCT levels rise rapidly, usually within 2–3 h, and peak around 12–24 h after infection onset, with a half-life of approximately 22–35 h [36,37,38,39,40,41,42,43,44,45,46,47,48,49,50,51,52,53,54]. These characteristics make it particularly suitable for early detection and monitoring of infectious processes. Prior studies have demonstrated the usefulness of PCT in diagnosing postoperative sepsis [40], guiding antibiotic stewardship [35,41], and predicting complications following major gastrointestinal procedures such as colorectal surgery, esophagectomy, and pancreaticoduodenectomy [42,43,44,45]. It has also shown utility in identifying abscesses in chronic inflammatory conditions like Crohn’s disease [46].

The role of PCT in acute appendicitis remains debated, with some studies supporting its diagnostic utility [47,48,49], while others report limited sensitivity in early identification of appendiceal inflammation [50,51]. More recently, evidence has emerged indicating that elevated preoperative PCT may differentiate complicated from uncomplicated appendicitis [52,53]. However, until now, no studies have specifically evaluated whether PCT trends can predict post-appendectomy complications in patients with uncomplicated disease. In our study, patients who developed post-operative abscesses had consistently higher PCT levels at all measured time points—preoperatively, immediately after surgery, on the first post-operative day, and at discharge—compared to patients without complications. While both groups exhibited a decline in PCT following surgery, the kinetics differed: the abscess group showed a sharper drop immediately after the procedure, followed by a plateau or slight increase, whereas in the control group, PCT levels declined steadily and consistently. These findings suggest that both absolute PCT values and their dynamic trajectory over time may provide important prognostic insights. One of the most significant aspects of this study is the integration of point-of-care testing (POCT) for PCT. Unlike traditional laboratory assays that may require several hours, POCT devices can deliver results within 30 min, making them highly applicable in emergency and perioperative settings [55,56,57,58,59,60,61]. Rapid availability of PCT values may facilitate timely decisions regarding imaging, escalation of antibiotics, or the need for closer monitoring, especially in patients showing atypical clinical evolution. Moreover, PCT-guided antibiotic protocols have been associated with shorter treatment durations and reduced healthcare costs without compromising clinical outcomes [62,63].

From a healthcare perspective, the development of post-operative abscesses necessitates re-hospitalizations, prolonged antibiotic therapy, and invasive procedures, all of which increase patient morbidity and overall system burden. Early identification of high-risk individuals through PCT monitoring could reduce these downstream consequences by enabling targeted interventions. Although POCT adds modest cost per test (€10.00 vs. €13.00 for lab-based assays), its potential to prevent costly complications justifies further investigation into its routine adoption. Nonetheless, the feasibility of universal PCT testing in all appendectomy patients should be balanced against more selective use based on clinical or intraoperative findings.

This study has several limitations. As a pilot analysis with a relatively small sample size (33 patients), our findings should be interpreted with caution. The statistical power was limited, particularly for subgroup analyses, and further multicenter studies are required to validate these preliminary observations. Moreover, while the trends in PCT kinetics were promising, their precise role as a standalone or adjunctive predictor needs to be clarified through prospective validation. Future research should also address cost-effectiveness models, patient-centered outcomes, and the development of clinical algorithms integrating PCT with other risk factors to guide post-operative care after appendectomy.

## 5. Conclusions

This pilot study provides compelling evidence that PCT, both in terms of absolute levels and kinetics, may serve as a valuable early marker for predicting the development of post-operative abscesses in patients undergoing appendectomy for non-complicated acute appendicitis. The integration of POCT for PCT measurement into clinical practice holds promise for improving patient outcomes by enabling earlier detection and intervention in high-risk cases. Further research is warranted to validate these findings in larger cohorts and to explore the cost-effectiveness of routine PCT monitoring in surgical settings. PCT kinetics, whether measured through POCT or traditional methods, show promise as an early indicator for post-operative abscess formation in patients with appendicitis. Further studies with larger populations are necessary to confirm these findings and validate the utility of POCT for this purpose.

## Figures and Tables

**Table 1:** Clinical and laboratory data in all patients.

Variables	N. of Patients	%
Age, year	36.0 ± 17.9	-
Male Sex	17	51.5
Blumberg’s sign	13	39.4
Pain migration	5	15.1
Symptom duration > 48 h	9	27.3
Comorbidities	16	48.5
Diabetes	5	15.1
Morbidity rate	9	27.3
Creatinine mg/dL	0.7 ± 0.2	-
Glycemia mg/dL	109.0 ± 58.0	-
Lactate Dehydrogenase H UI/L	197.0 ± 100.0	-
C-reactive protein mg/L	49.9 ± 59.0	-
Bilirubin mg/dL	1.0 ± 0.7	-
White blood cells count ×10^9^/L	12.4 ± 4.1	-
Platelets ×10^9^/L	233.0 ± 68.0	-

**Table 2:** PCT values in all patients.

Time	PCT ng/mL
T0	2.03 ± 3.89
T1	0.70 ± 1.51
T2	0.90 ± 1.05
T3	0.41 ± 0.37

**Table 3:** Comparison of demographic, clinical and laboratory data between the two-study group.

Variables ^1^	Group 1	Group 2	*p* Value
N. of patients	4	29	-
Age, year	46.0 ± 31.0	35.0 ± 15.7	0.02
Male Sex	3	13	0.3
Blumberg’s sign	2	11	0.6
Pain migration	2	3	0.5
Comorbidities	1	15	0.6
Diabetes	1	4	0.7
Morbidity rate	1	8	0.5
Creatinine mg/dL	0.9 ± 0.5	0.7 ± 0.2	0.2
Glycemia mg/dL	114.3 ± 22.4	108.2 ± 61.1	0.8
LDH UI/L	273.5 ± 170.4	190.4 ± 95.0	0.2
CRP mg/L	93.3 ± 94.0	44.6 ± 54.0	0.1
Bilirubin mg/dL	1.6 ± 0.8	0.9 ± 0.7	0.09
WBC ×10^9^/L	13.4 ± 2.2	12.4 ± 4.3	0.6
PLT ×10^9^/L	192.0 ± 126.0	237.5 ± 61.4	0.2
Minimally invasive surgery	1	0	0.6

^1^ CRP: C-reactive protein; LDH: Lactate dehydrogenase; WBC: White blood count cells; PLT: platelets.

**Table 4:** Comparison of the decrease in PCT values between the two-study group.

Variables	Group 1	Group 2
N. of patients	4	29
T0 vs. T1	−49.15%	−47.37%
T1 vs. T2	−6.04%	−53.33%
T2 vs. T3	−53.57%	−7.14%

## Data Availability

All the data used are present in the text. No additional data are available.

## Abbreviations

The following abbreviations are used in this manuscript:

CRP	C-reactive protein
PCT	Procalcitonin
POCT	Point-of-care testing
LDH	Lactate dehydrogenase
PLT	Platelets
WBC	White blood cells
